# miR-185 inhibits prostate cancer angiogenesis induced by the nodal/ALK4 pathway

**DOI:** 10.1186/s12894-020-00617-2

**Published:** 2020-05-04

**Authors:** Youkong Li, Wen Zhong, Min Zhu, Mengbo Li, Zhenwei Yang

**Affiliations:** 1 Department of Urology, Jingzhou Central Hospital and The Second Clinical Medical College, Yangtze University, No.60 Jingzhong Road, Jingzhou District, Jingzhou, 434020 Hubei Province People’s Republic of China; 2 Department of Endocrine, Jingzhou Central Hospital and The Second Clinical Medical College, Yangtze University, Jingzhou, 434020 Hubei Province People’s Republic of China

**Keywords:** Nodal, miR-185, ALK4, Angiogenesis, Prostate cancer

## Abstract

**Background:**

Inhibition of angiogenesis in prostatic cancer could be a brand-new method to suppress tumour progression. Nodal/ALK4 has been associated with vascularization in many cancers. However, the relationship between and role of Nodal/ALK4 and miR-185 in human prostatic cancer is still unknown.

**Methods:**

Prostatic cancer DU145 cells and LNCaP cells were used to investigate the angiogenic effect induced by Nodal and the anti-angiogenic roles of miR-185. Colony formation assay, MTT assay, transwell assay and tube formation assay were used to explore cell proliferation, migration and tube-forming ability, respectively. A luciferase reporter assay confirmed the binding relationship between miR-185 and ALK4. The expression levels of miR-185, ALK4 and VEGF were detected by qRT-PCR and Western blotting. The effects of miR-185 and Nodal in prostate cancer were also investigated in animal experiments.

**Results:**

VEGF expression was increased in DU145 cells and LNCaP cells after Nodal incubation, and Nodal activated the proliferation ability of prostatic cancer cells and the migration and tube-forming ability of human umbilical vein endothelial cells (HUVECs), which were all inhibited by treatment with the Nodal inhibitor SB431524. Bioinformatics analysis and luciferase assay were used to verify miR-185 as a target of ALK4. Prostatic cancer cell proliferation was inhibited by overexpression of miR-185, which was shown to regulate the migration and angiogenesis of HUVECs by targeting ALK4 for suppression. miR-185 also showed a significant inverse correlation with Nodal treatment and reversed the angiogenic effects induced by Nodal. More importantly, for the first time, xenograft experiments indicated that overexpression of miR-185 suppressed tumour development.

**Conclusion:**

The Nodal/ALK4 pathway is important in the angiogenesis of prostate cancer and can be inhibited by targeting miR-185 to downregulate ALK4. These findings provide a new perspective on the mechanism of prostate cancer formation.

## Background

Prostate cancer is the most common malignant tumour among men and has the second highest mortality rate among cancers in developed countries and regions such as Europe and the United States [1]. The incidence of prostate cancer in Asia is lower than that in Europe, but it has shown a rapid upward trend in recent years [2]. The blood supply of malignant tumours is a key factor in tumour growth. It is known that some tumour cells play a role in the angiogenesis pathway to provide sufficient oxygen and nutrients for tumour growth, invasion and metastasis and have different characteristics than conventional vascular endothelial cells [3]. Inhibition of tumour vascularization has been found to increase the necrosis of tumour cells. Thus, in-depth exploration of the molecular mechanism of angiogenesis in prostate cancer is of great significance for its treatment.

Nodal, a member of the transforming growth factor beta (TGFβ) superfamily, has properties that promote tumour cell plasticity and tumourigenicity [4, 5]. It was reported in the literature that Nodal was highly expressed in prostate cancer cells such as WPE, DU145, and LNCaP and could regulate prostate cancer proliferation by activating smad 2/3 phosphorylation by activin receptor-like kinase 4 (ALK4) [6]. Another report indicated that Nodal could promote the formation of tumour angiogenesis mimicry in malignant tumours such as breast cancer [7]. However, whether the Nodal/ALK4 pathway is related to the regulation of prostate cancer angiogenesis has not been reported in the literature.

In recent years, miRNAs have been shown to have an important effect on the progression of tumours, especially by acting as oncogenes or tumour suppressor genes in tumourigenesis and the regulation of tumour molecular mutations. Each miRNA has many potential target genes, and finding a true target gene that acts on tumour cell behaviour is crucial. miR-185 was shown to be expressed at low levels in prostate cancer cell lines and tissues and to inhibit prostate cancer progression by inducing apoptosis [8]. A previous study demonstrated that in human microvascular endothelial cells, miR-185 could inhibit angiogenesis by targeting STIM1 [9], but the function of miR-185 in prostate cancer angiogenesis has not been reported. We found that there was a binding site for miR-185 on ALK4 through bioinformatics analysis. Therefore, we wanted to test whether miR-185 inhibited the Nodal/ALK4 pathway, thereby inhibiting prostate cancer angiogenesis.

In this study, the relationship between the Nodal/ALK4 pathway and angiogenesis was first evaluated in prostate cancer cells. Then, whether miR-185 could regulate the Nodal/ALK4 pathway was investigated. The anti-angiogenic effects of miR-185 in prostate cancer cells were explored via an in vitro cell model and with animal experiments. Therefore, our results could determine whether miR-185 inhibited the Nodal/ALK4 pathway and angiogenesis in prostate cancer.

## Methods

### Cell culture and treatment

Human umbilical vein endothelial cells (HUVECs) and the human prostate cancer DU145 cells and LNCaP cells were purchased from American Type Culture Collection (ATCC, Manassas, VA, USA). RPMI 1460 medium (Gibco, Waltham, MA, USA) supplemented with 10% foetal bovine serum (HyClone, Logan, UT) and 1% penicillin–streptomycin (Gibco, Waltham, MA, USA) was used for cell culture at 37 °C in 7% CO_2_ for 3–4 days. To study the relationship between the Nodal/ALK4 pathway and angiogenesis, both cell lines were treated with Nodal (Sigma, St Louis, MO, USA) for 8 h at a concentration of 5 μM. To explore the effect of prostate cancer on angiogenesis, HUVECs were co-cultured with DU145 cells and LNCaP cells.

### Quantitative reverse-transcription polymerase chain reaction (qRT-PCR)

miR-185 was verified using a TaqMan primer and probe set from Applied Biosystems. Total RNA was isolated from human DU145 cells and LNCaP cells using TRIzol reagent (TransGen Biotechnology Co., Ltd., Beijing, China). A cDNA Reverse Transcription kit (TransGen Biotechnology Co., Ltd., Beijing, China) was used to obtain cDNA following the manufacturer’s instructions. Single-stranded cDNA was generated by TaqMan PCR Master Mix (TransGen Biotechnology Co., Ltd., Beijing, China), and U6 and GAPDH acted as internal controls. The primers used for the analysis were as follows: miR-185 forward: 5′-CGCTGGAGAGAAAGGCAGT-3′, reverse: 5′-GTCGTATCCAGTGCAGGGTCCGAGGTATTCGCACTGGATACGACTCAGGA-3′; ALK4 forward: 5′-CTGCAACAGGATCGACTTGA-3′, reverse: 5′-GGAGCGTCTTGTCTTTGGAG-3′; VEGF forward: 5′-GGGCAGAATCATCACGAAGT-3′, reverse: 5′- TGGTGATGTTGGACTCCTCA-3′; U6 forward: 5′-CTCGCTTCGGCAGCACA-3′, reverse: 5′-AACGCTTCACGAATTTGCGT-3′; GAPDH forward: 5′-TGTGGGCATCAATGGATTTGG-3′, reverse: 5′-ACACCATGTATTCCGGGTCAAT-3′. PCRs were run three times, and the experiment was conducted in triplicate. According to the formula 2^-ΔΔCt^, the relative fold changes in mRNA expression were verified [10].

### Western blot analysis

Total proteins were extracted from tissues and cells by RIPA buffer, and the concentration was determined by a BCA protein assay kit. Protein (30 μg) was separated by 10% sodium dodecyl sulfate (SDS) polyacrylamide gel electrophoresis (PAGE) and transferred onto polyvinylidene fluoride (PVDF) membranes (Santa Cruz Biotech, USA). After blocking with 15% blocking solution, the membranes were incubated with a primary rabbit polyclonal antibody against VEGF (1:800, Abcam, Cambridge, MA, USA) and primary mouse monoclonal antibody against ALK4 (1:500, Santa Cruz Biotechnology, Inc., Dallas, TX, USA) overnight at 4 °C. A primary rabbit polyclonal antibody against β-actin (1:2000, Abcam, Cambridge, MA, USA) was then applied as an internal reference. Following three washes with TBST, a horseradish peroxidase-conjugated antibody against the corresponding species (1:2000, Santa Cruz Biotech, USA) was added for a 1 h incubation at room temperature, and then an ECL detection kit (Jiancheng Bioengineering Institute, Nanjing, China) was used for band detection. All bands were visualized and quantified by ImageJ software (NIH, Bethesda, Maryland, USA). The experiment was conducted in triplicate. The result was expressed as the band intensity ratio of the target protein to the internal reference protein.

### Colony formation assay

After 14 days of culture, the corresponding treated DU145 cells and LNCaP cells were fixed with methanol. Crystal violet dye solution was used to stain colonies, and then the colony-forming units were counted under an optical microscope (Leica, Wetzlar, Germany) with a 20-fold objective.

### MTT assay

Cell viability was assessed by the MTT assay. Human prostate cancer DU145 cells and LNCaP cells were co-cultured with HUVECs and then subjected to specific treatment, which included Nodal or Nodal+SB431524 incubation for 8 h, or miR-185 mimics or miR-NC transfection. After treatment, MTT (0.5 mg/ml in pH 7.4 PBS, Sigma, USA) was added to each well for 4 h at 37 °C. The supernatant was removed. After washing with phosphate-buffered saline (PBS) three times, 150 μl dimethyl sulfoxide (DMSO, Sigma, USA) was added, and the plate was agitated to dissolve the precipitates. The absorbance was measured by a multiwell spectrophotometer (Biotek Instruments Inc., Burlington, Vermont) at 490 nm. The experiment was conducted in triplicate.

### Transwell assay

In 8-mm pore 24-well transwell chambers, HUVECs were first cultured for 8 h before invasion towards the conditioned medium. The upper chamber was filled with 200 μl serum-free medium, while the lower chamber contained 10% FBS medium. After 48 h of incubation, cells in the upper chamber were collected with a cotton swab. Then, cells were fixed with 5% glutaraldehyde in PBS for 15 min, and 0.1% crystal violet was applied to the lower chamber for microscopic inspection (Leica, Wetzlar, Germany). At least five different fields were randomly selected for each sample. The cell migration capacity was verified by calculating the number of cells passing through the membrane.

### Tube formation assay

Basement membrane matrix (BMM) was added to each well and then allowed to harden at 37 °C for 30 min. HUVECs were cultured on top of the BMM-coated wells for 48 h at 37 °C. The morphology of HUVECs was observed under an optical microscope (Leica, Wetzlar, Germany) with a 20-fold objective. Images were obtained using an Olympus IX70 microscope (Tokyo, Japan).

### Cell transfection

Human miR-185 mimics and their relative control mimics (mimics NC) and human miR-185 inhibitor and its relative control inhibitor (inhibitor NC) were purchased from GenePharma (Shanghai, China). Full-length human ALK4 cDNA was amplified and cloned into the pcDNA3.1 vector (OE-ALK4). DU145 cells or LNCaP cells were cultured, and Lipofectamine 2000 (Invitrogen, California, USA) containing nucleotide fragments was added to Opti-MEM at room temperature for a 0.5-h incubation and then added to cells for a 6-h incubation. After removing the above culture medium, cells were cultured under normal conditions for detection after 48 h.

### Luciferase activity assay

To obtain the reporter vector ALK4-wild-type, the fragment from the 3′-untranslated region (3’UTR) of ALK4 containing the predicted miR-185 binding site was synthesized and cloned into the pmirGLO vector (Promega, Madison, WI, USA). Sites were mutated to generate ALK4-mutated to disrupt the predicted miR-185 binding site. miR-185 mimics or miR-185 inhibitor and vectors (WT-ALK4–3’UTR or MUT-ALK4–3’UTR) were cotransfected into cells. After 48 h, cells were analysed by the dual-luciferase kit (Promega, California, USA) for luciferase activity. Each experiment was performed in triplicate. The results are presented as relative luciferase activity (firefly LUC/Renilla LUC).

### Xenograft experiments in nude mice

All animal experiments were performed in accordance with protocols approved by the Animal Research Committee of the Second Clinical Medical College of Yangtze University. Male BALB/c nude mice (approximately 20 g) at 6 weeks of age were purchased from SJA Laboratory Animal Co., Ltd. (Hunan, China). DU145 cells (2 × 10^6^) and LNCaP cells (2 × 10^6^) were subcutaneously injected into the right side of the armpits of BALB/c nude mice. To assess the antitumour activity in a xenograft model in vivo, the mice were randomly divided into four groups: control, Nodal, mimics NC, and miR-185 mimics (*n* = 6 per group). After palpable tumours developed a volume of approximately 50 mm^3^, the mice were injected with a PBS solution as a control, Nodal, mimics NC, or miR-185 mimics through intratumor injection, and tumour growth was examined during the course of 28 days. The tumour width and length were measured by a micrometre. The tumour volumes were calculated as (length × width^2^)/2. Tumour growth was observed for 28 days, at which time the mice were euthanized. The largest diameters of the formed tumours were measured macroscopically, and the tumours were weighed with an electronic balance at the end of the experiment.

### Immunohistochemical staining

Tumour tissues were obtained from euthanized mice after the indicated treatments and fixed with paraformaldehyde (Sigma-Aldrich, St Louis, MO, USA) diluted to 10% in PBS for 24 h. For immunohistochemical staining, the sections were cut serially (5 μm) and boiled at 121 °C for 20 min in 10 mM citrate buffer solution (pH 6.0) for antigen retrieval. After endogenous peroxidase activity was blocked with 0.3% hydrogen peroxide, the sections were incubated with a primary rabbit polyclonal antibody against CD31 (1:800, Abcam, Cambridge, MA, USA) overnight at 4 °C. After adding a goat anti-rabbit secondary antibody (1:1200, Abcam, Cambridge, MA, USA) for 30 min incubation at room temperature, the sections were incubated with a DAB detection kit (Jiancheng Bioengineering Institute, Nanjing, China). Following dehydration and clearing, the sections were analysed by general microscopy (Leica, Wetzlar, Germany).

### Statistical analysis

Statistical analysis of the data was performed in SPSS 16.0. All values are shown as the average ± SD. Student’s t-test and one-way analysis of variance (ANOVA) were used to perform comparisons between two groups and multiple groups, respectively. Statistically significant differences were considered at *P* < 0.05.

## Results

### Nodal promoted prostate cancer cell-induced angiogenesis

**Fig. 1:**
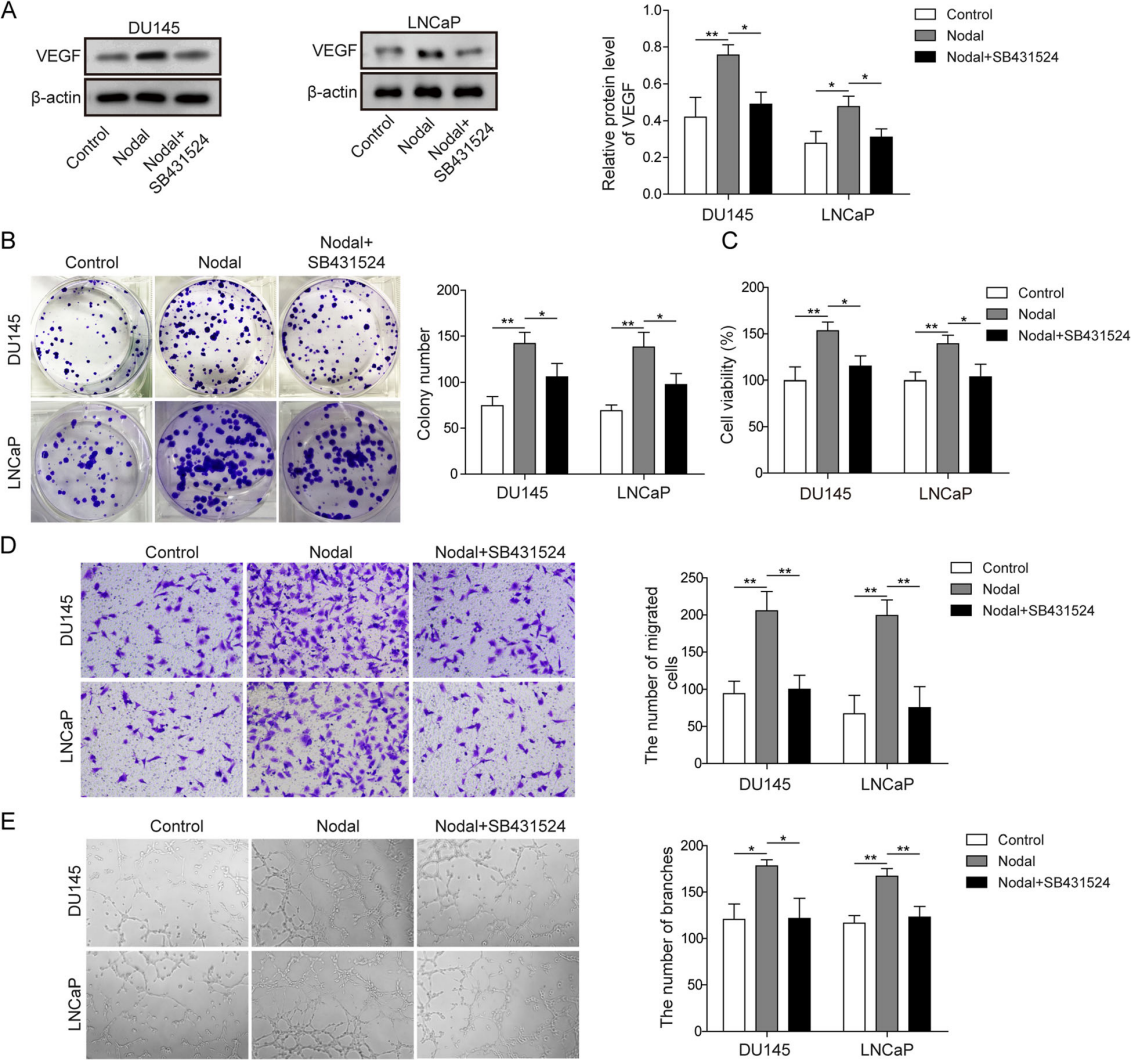
Nodal promoted prostate cancer cell-induced angiogenesis. **a** Protein levels of VEGF were assessed by Western blot in DU145 cells and LNCaP cells treated with Nodal or SB431524. **b** Representative photomicrographs of the colony formation assay and quantification of colony numbers in DU145 cells and LNCaP cells treated with Nodal or SB431524. **c** Cell viability of co-cultured HUVECs was evaluated by MTT assay and was determined as the percentage of the value relative to that of the control group. **d** The migration ability of co-cultured HUVECs was detected by transwell assay. **e** Tube forming ability of HUVECs was observed after the indicated treatment. The data represent one of three independent experiments. Error bars denote the mean ± SD. *P < 0.05, **P < 0.01

To determine whether Nodal treatment affected prostate cancer cell angiogenesis, DU145 cells and LNCaP cells were divided into three groups: control, Nodal, and Nodal+SB431524, where SB431524 is an inhibitor of Nodal. Western blot analysis of VEGF in DU145 cells and LNCaP cells was first performed. The results are shown in Fig. 1a. This finding suggested that Nodal induced VEGF expression in both DU145 cells and LNCaP cells, which was inhibited by SB431524. Then, a colony formation assay was used to detect the proliferation of prostate cancer cells. The results in the two cell types consistently demonstrated that the proliferative capacity of the Nodal group was stronger than that of the control group, while there was no significant difference between the control group and the Nodal+SB431524 group (Fig. 1b). The results of cell viability at 8 h after incubation with Nodal or Nodal+SB431524 in HUVECs co-cultured with DU145 cells or LNCaP cells were consistent with the colony formation assay results detected by MTT (Fig. 1c). Additionally, the migration ability of HUVECs co-cultured with DU145 cells or LNCaP cells was detected by transwell assay. Nodal induced HUVEC migration in co-culture with DU145 cells or LNCaP cells. Compared to DU145 cells or LNCaP cells treated with Nodal alone, HUVECs co-cultured with DU145 cells or LNCaP cells decreased the number of migrated HUVECs after treatment with Nodal+SB431524 (Fig. 1d). Angiogenesis mimicry in co-culture of HUVECs with DU145 cells or LNCaP cells in three-dimensional culture was observed. Slight angiogenesis mimicry was observed in the control group at 48 h in the three-dimensional culture, but obvious angiogenesis mimicry was observed in the Nodal group (Fig. 1e). By adding SB431524, the observed angiogenesis mimicry induced by Nodal was relieved (Fig. 1e). These results indicated that Nodal promoted prostate cancer cell-induced angiogenesis.

### miR-185 inhibited ALK4 expression by binding to the 3′-UTR of ALK4

**Fig. 2:**
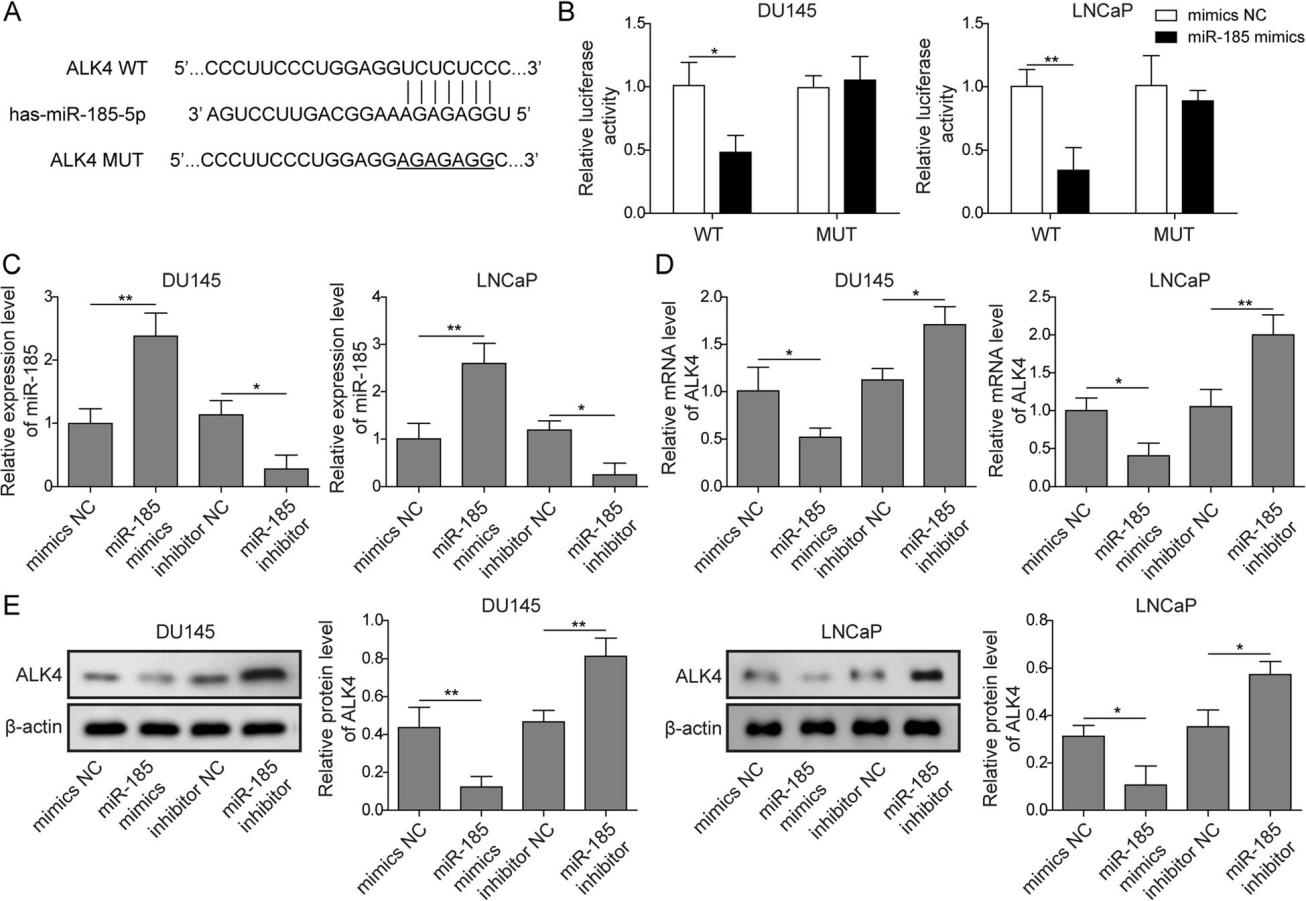
miR-185 inhibited ALK4 expression by binding to the 3′-UTR of ALK4. **a** Schematic of the candidate miRNAs that target ALK4 predicted by StarBase. **b** Dual-luciferase assay was performed to study the interaction between miR-185 and ALK4. The expression levels of miR-185 **c** and ALK4 **d** were quantified by qRT-PCR in DU145 cells and LNCaP cells transfected with miR-185 mimics or miR-185 inhibitor. **e** Protein levels of ALK4 were assessed by Western blotting in DU145 cells or LNCaP cells with the indicated treatments. The data represent one of three independent experiments. Error bars denote the mean ± SD. *P < 0.05, **P < 0.01

By using StarBase, we predicted the miRNAs that may interact with ALK4 to investigate interactions between ALK4 and miRNAs. The bioinformatics prediction results shown in Fig. 2a indicate the presence of a binding site of miR-185 on ALK4. We also performed a luciferase reporter assay to further verify whether ALK4 was a functional target of miR-185. The results demonstrated that miR-185 mimics decreased the luciferase activity of ALK4-WT, whereas it had no influence on the luciferase activity of ALK4-MUT (Fig. 2b). qRT-PCR (Fig. 2c&d) and Western blotting (Fig. 2e) were performed to verify the expression levels of miR-185 and ALK4. In DU145 cells and LNCaP cells, miR-185 inhibitor decreased the gene expression level of miR-185 and consequently increased the gene and protein expression of ALK4, while miR-185 mimics increased the gene expression level of miR-185 and thus decreased the gene and protein expression of ALK4. The Western blot and qRT-PCR results were consistent and suggested a potential role of miR-185 in regulating ALK4 expression.

### miR-185 targets ALK4 to regulate angiogenesis induced by prostate cancer cells

**Fig. 3:**
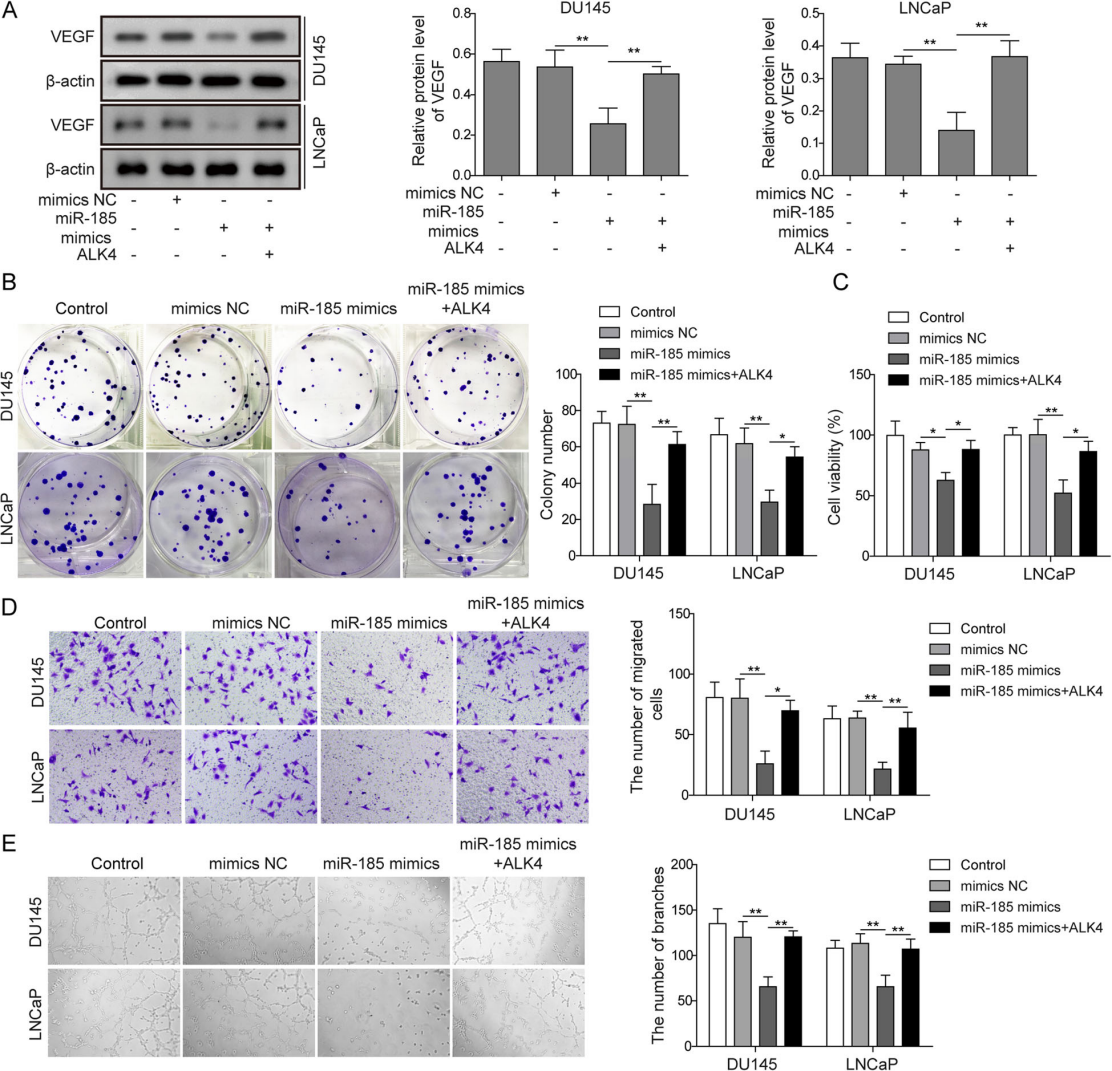
MiR-185 targeted ALK4 to regulate angiogenesis induced by prostate cancer cells. **a** Protein levels of VEGF were assessed by Western blot in DU145 cells and LNCaP cells transfected with miR-185 mimics or pcDNA3.1-ALK4. **b** Representative photomicrographs of the colony formation assay and quantification of colony numbers in DU145 cells and LNCaP cells transfected with miR-185 mimics or pcDNA3.1-ALK4. **c** Cell viability of co-cultured HUVECs was evaluated by MTT assay and was determined as the percentage of the value relative to that of the control group. **d** The migration ability of co-cultured HUVECs was detected by transwell assay. **e** Tube forming ability of HUVECs was observed after the indicated treatment. The data represent one of three independent experiments. Error bars denote the mean ± SD. *P < 0.05, **P < 0.01

We have demonstrated the relationship between miR-185 and ALK4. To further prove whether miR-185 could regulate angiogenesis through ALK4, DU145 cells or LNCaP cells were divided into four groups: control, mimics NC, miR-185 mimics, and miR-185 mimics+OE-ALK4. Western blot analysis of VEGF in DU145 cells and LNCaP cells was first performed (Fig. 3a). miR-185 mimics significantly inhibited VEGF expression compared with that in the control group and mimics NC group, while overexpression of ALK4 reversed the above effect of miR-185 mimics (Fig. 3a). The colony formation assay shown in Fig. 3b clearly demonstrated that miR-185 mimics inhibited prostate cancer cell proliferation, which was blocked by ALK4 overexpression. The viability of HUVECs co-cultured with DU145 cells or LNCaP cells displayed the same trends (Fig. 3c). With miR-185 mimic treatment, HUVEC viability was reduced, and simultaneous overexpression of miR-185 and ALK4 could restore viability to the level of the control group (Fig. 3c). Cell migration was inhibited in HUVECs co-cultured with DU145 cells or LNCaP cells treated with miR-185 mimics, while miR-185 mimics plus OE-ALK4 increased the number of migrated HUVECs (Fig. 3d). Additionally, Fig. 3e verified that miR-185 mimics + OE-ALK4 reversed HUVEC angiogenesis inhibited by miR-185 mimics. Undoubtedly, ALK4 is regulated by miR-185, and thus prostate cancer cell angiogenesis could be affected.

### miR-185 inhibited angiogenesis through the nodal/ALK4 pathway

**Fig. 4:**
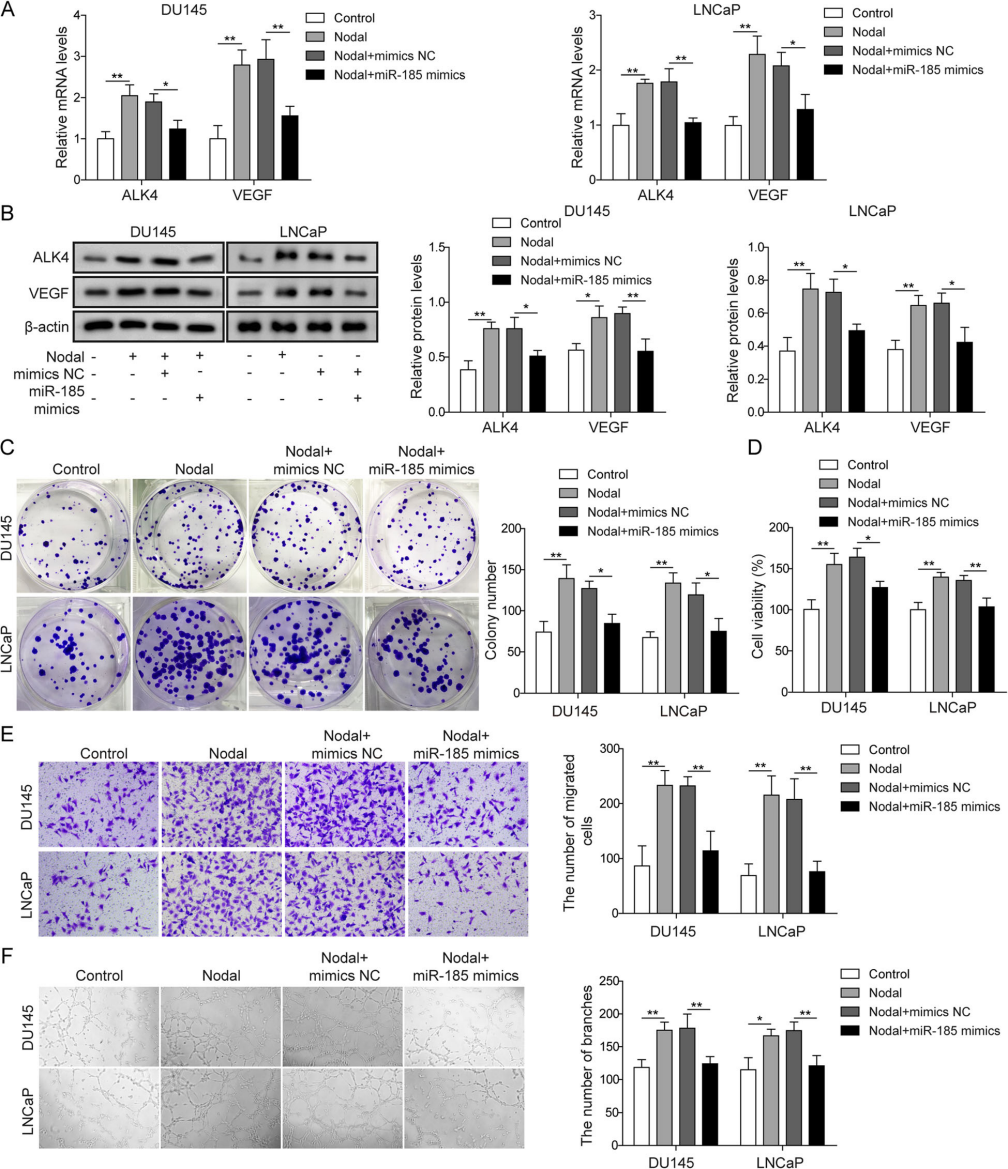
miR-185 inhibited angiogenesis through the Nodal/ALK4 pathway. **a** The mRNA levels of ALK4 and VEGF were quantified by qRT-PCR in DU145 cells and LNCaP cells treated with Nodal or transfected with miR-185 mimics. **b** Protein levels of ALK4 and VEGF were assessed by Western blot in DU145 cells and LNCaP cells with the indicated treatments. **c** Representative photomicrographs of the colony formation assay and quantification of colony numbers in DU145 cells and LNCaP cells treated with Nodal or transfected with miR-185 mimics. **d** Cell viability of co-cultured HUVECs was evaluated by MTT assay and was determined as the percentage of the value relative to that of the control group. **e** The migration ability of co-cultured HUVECs was detected by transwell assay. **f** The tube forming ability of HUVECs was observed after the indicated treatment. The data represent one of three independent experiments. Error bars denote the mean ± SD. *P < 0.05, **P < 0.01

To explore the role of miR-185 in Nodal/ALK4 pathway-induced angiogenesis, DU145 cells or LNCaP cells were divided into four groups: control, Nodal, Nodal+mimics NC, and Nodal+miR-185 mimics. qRT-PCR (Fig. 4a) and Western blotting (Fig. 4b) of ALK4 and VEGF in DU145 cells and LNCaP cells were first conducted. The results of qRT-PCR and Western blot consistently suggested the potential role of miR-185 mimics in regulating the Nodal/ALK4 signalling pathway. Nodal treatment induced ALK4 expression, thereby increasing VEGF levels (Fig. 4a&b). However, with miR-185 mimic treatment, the expression of ALK4 was significantly decreased compared with that in the Nodal+mimics NC group, thus reversing the level of VEGF (Fig. 4a&b). The colony formation assay results showed that treatment with Nodal alone induced prostate cancer cell proliferation, and miR-185 mimics inhibited the above effect of Nodal (Fig. 4c). In HUVECs co-cultured with DU145 cells or LNCaP cells, Nodal induced HUVEC proliferation, and the viability of HUVECs was higher than that of the control group, which was restored by miR-185 mimics (Fig. 4d). The migration ability results in HUVECs was confirmed with the cell viability results in HUVECs (Fig. 4e), displaying the opposite effect between Nodal and miR-185 mimics. In the angiogenesis test of HUVECs co-cultured with DU145 cells or LNCaP cells, Nodal induced HUVEC angiogenesis, and miR-185 mimics inhibited the angiogenic effect of Nodal (Fig. 4f). Therefore, miR-185 showed a significant inverse correlation with Nodal treatment, reversing the angiogenesis effects induced by Nodal.

### Overexpression of miR-185 exerted antitumor activity against tumor growth in nude mice

**Fig. 5:**
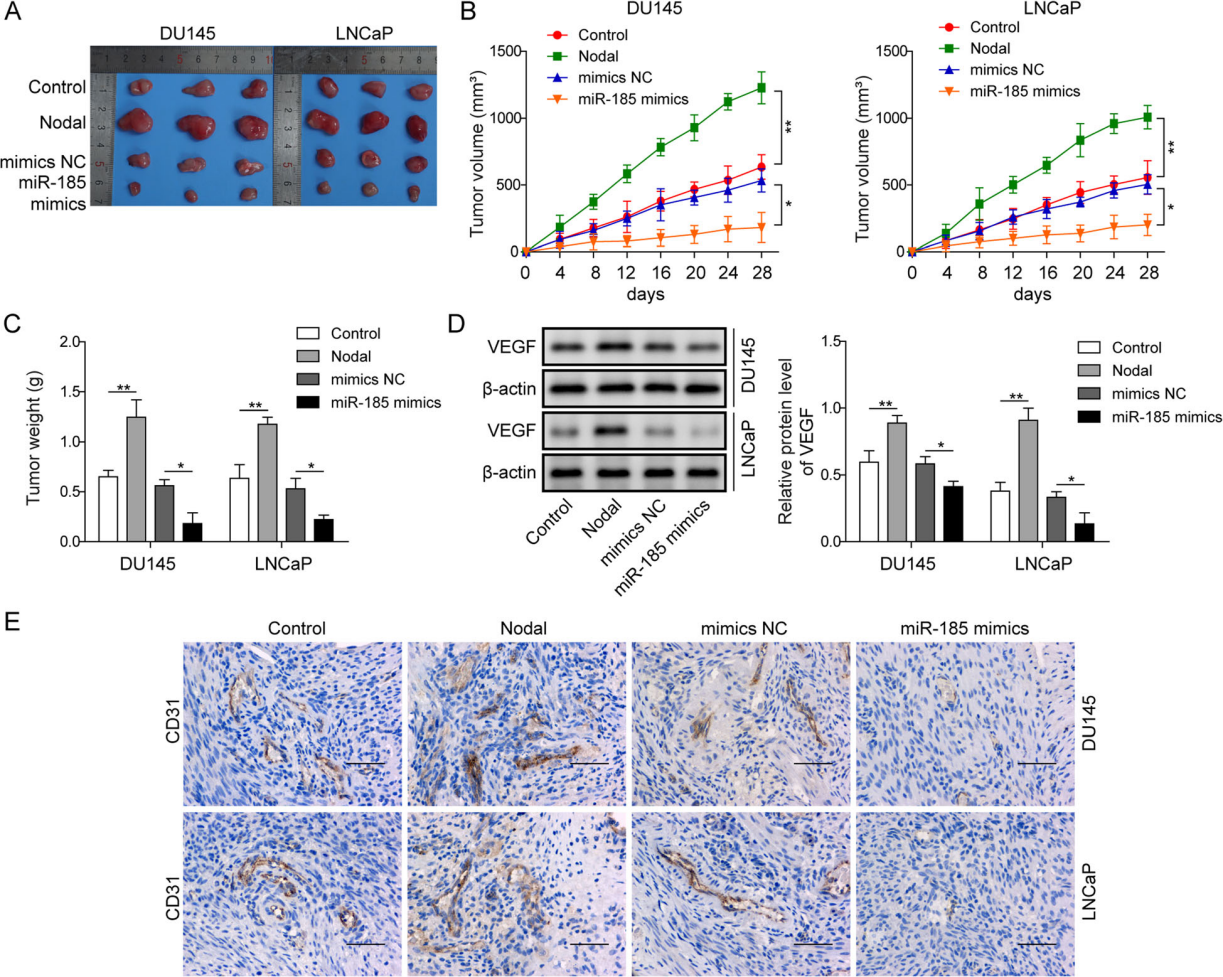
Overexpression of miR-185 exerted antitumour activity against tumour growth in nude mice. **a** The macroscopic appearance of the dissected tumour tissues 30 days after the treatments. **b** The tumour size was calculated in nude mice subjected to specific treatments. **c** The tumour weight was calculated in nude mice subjected to specific treatments. **d** Protein levels of VEGF were assessed by Western blot in nude mice treated with specific treatments. **e** The distribution of CD31 was detected by immunohistochemistry. Scale bar: 50 μM. The data represent one of three independent experiments. Error bars denote the mean ± SD. *P < 0.05, **P < 0.01

To determine the in vivo effects of miR-185 and Nodal on tumor growth in a xenograft model, DU145 cells or LNCaP cells were injected subcutaneously into 6-week-old male athymic nude mice (BALB/c-nu/nu). The mice were randomly divided into control, Nodal, mimics NC and miR-185 mimics group. The macroscopic appearance of the dissected tumor tissues after 28 days from specific treatments was shown in Fig. 5a. Obviously, the tumors in the Nodal-treated group were significantly larger than those in the control group, and after overexpressing miR-185, the tumors were the smallest. The statistical results of tumor size (Fig. 5b) and tumor weight (Fig. 5c) were consistent with the above trends. The results of Western blotting in Fig. 5c verified that Nodal treatment accelerated the expression of VEGF while overexpression of miR-185 inhibited this abnormal expression. Based on basic level of VEGF in control group, miR-185 overexpressed attenuated the tumor progression. As shown in Fig. 5e, there was significantly abundant expression of angiogenesis biomarker CD31 observed in Nodal group, while miR-185 mimics could reduce the expression of CD31. Analysis of the data of xenograft experiments revealed that overexpression of miR-185 suppressed tumor growth in an in vivo xenograft model.

## Discussion

In this study, we provided evidence of the association between Nodal and prostatic cancer. The results suggest that Nodal/ALK4 is involved in the regulation of angiogenesis in pancreatic cancer. miR-185 could decrease prostatic cancer cell proliferation and HUVEC migration and angiogenesis, while overexpression of ALK4 in DU145 cells or LNCaP cells could suppress the effect of miR-185, indicating that miR-185 targeted ALK4 to regulate angiogenesis in prostate cancer. The animal experiments clearly showed that overexpression of miR-185 suppressed prostate carcinoma development in vivo. All the results implied that miR-185 may act as a tumour suppressor in the angiogenesis of prostate cancer by inhibiting the Nodal/ALK4 pathway.

Angiogenesis is the basis of reproduction, growth and repair and is also a necessary condition for tumour growth and metastasis. Studies have shown that high protein expression in prostate cancer tissue can promote blood vessel growth [11]. In our study, we found that Nodal incubation could increase the expression of VEGF, resulting in HUVEC proliferation and migration, which is characteristic of vascularization. Nodal is a member of the transforming growth factor beta superfamily. It has been found that Nodal expression is limited to human embryonic stem cells and embryonic tissues [12]. However, the Nodal protein was found to be highly expressed in some tumour tissue cells, suggesting that Nodal expression is associated with tumourigenesis, development, invasion and metastasis. Taylor et al. found that the protein expression of Nodal was lower in benign prostatic disease tissues than in prostate cancer tissues [13]. Stable expression of the Nodal gene enhanced LNCaP cell growth on Matrigel [14], which is consistent with our findings in the migration assay. Nodal promoted tumour angiogenesis by increasing the expression of the VEGF receptor [15], and downregulation of Nodal inhibited the plasticity of tumour cells [4]. In the study by Dueng-Yuan Hueng, Nodal accelerated the angiogenesis and growth of human gliomas, including glioma cell proliferation, colony formation, VEGF secretion, and angiogenesis [16]. Our research obtained similar results in prostatic cancer cells, showing that Nodal dramatically increased HUVEC viability and tube-forming ability.

miR-185 is located on chromosome 22q11.21, and its precursor consists of 82 nucleotides [17]. Although there has been no in-depth research on its function, miR-185 is considered to be a tumour suppressor gene in a variety of tumours and inhibits the proliferation of lung cancer [18], breast cancer [19], and colon cancer [20], which may indicate diverse effects of miR-185 in inhibiting cell proliferation. In lung cancer cells, miR-185 inhibited the expression of multiple cyclins [21]. In our study, Nodal increased the expression of VEGF, while overexpressing miR-185 inhibited the concentration of VEGF. miR-185 inhibited VEGF expression and HUVEC viability, migration and tube-forming ability, thus resulting in a reduced trend of angiogenesis. To further explore the inhibition mechanism of miR-185 on the angiogenesis of prostate cancer cells, we found through bioinformatics analysis that ALK4 had a miR-185 binding site. Overexpression of ALK4 in the Nodal pathway could significantly reverse the effects of miR-185 overexpression on VEGF levels, cell proliferation, migration and angiogenesis, but it could not be reversed to the same level as the control group, indicating that miR-185 exerted its antitumour effect through the Nodal/ALK4 pathway. In addition to Nodal/ALK4, miR-185 might exert its antitumour effect through other signalling pathways. It was demonstrated that miR-185 could suppress Wnt/β-catenin signalling and modulate the transcription and translation levels of downstream molecules of this pathway, including MYC and CCND1, in human colorectal cancer, thus exerting tumour suppressor effects [22]. Pan et al. found that lncAGER had an inhibitory effect on cell proliferation through upregulation of advanced glycosylation end-product specific receptor (AGER) by competitively binding to miR-185 in lung cancer [23]. It is possible that miR-185 inhibits cell proliferation by different mechanisms in different tumour cells. Zhu et al. found that miR-185 targeted Six2 and inhibited cell proliferation and epithelial-mesenchymal transition in hepatocellular carcinoma [24]. Another study showed that miR-185 inhibited angiogenesis in human microvascular endothelial cells by targeting stromal interaction molecule 1 [25]. Moreover, our results showed that miR-185 not only inhibited the proliferation of prostate cancer cells but also inhibited the migration and tube formation of HUVECs by inhibiting the Nodal/ALK4 pathway. Qu et al. found that miR-185 regulated cell cycle arrest at the G0/G1 phase through CDC6 to prevent the progression of prostate cancer [26]. Additionally, Li et al. reported that miR-185 is involved in prostate cancer through blockade of the SREBP metabolic pathway [27]. Therefore, we suspect that miR-185 may have multiple effects in prostate cancer, but in-depth studies are still needed to verify this assumption.

## Conclusions

In recent years, research on Nodal protein has become a new trend. At present, there are no reports on the expression and target significance of Nodal/ALK4 in prostate cancer cells. The results of this experiment showed that miR-185 could regulate Nodal/ALK4 pathway to inhibit the angiogenesis of prostate cancer, which provided a new perspective on the mechanism of prostate cancer formation.

## Data Availability

All data generated or analysed during this study are included in this published article.

## Abbreviations

ALK4: Activin receptor-like kinase 4
GADPH: Glyceraldehyde-3-phosphate dehydrogenase
HUVEC: Human umbilical vein endothelial cells
qRT-PCR: Quantitative real-time polymerase chain reaction
STIM1: Stromal interaction molecule 1
TGFβ: Transforming growth factor β
VEGF: Vascular endothelial growth factor
